# Identifying Common Genetic Risk Factors of Diabetic Neuropathies

**DOI:** 10.3389/fendo.2015.00088

**Published:** 2015-05-28

**Authors:** Ini-Isabée Witzel, Herbert F. Jelinek, Kinda Khalaf, Sungmun Lee, Ahsan H. Khandoker, Habiba Alsafar

**Affiliations:** ^1^Biomedical Engineering Department, Khalifa University of Science, Technology and Research, Abu Dhabi, United Arab Emirates; ^2^Australian School of Advanced Medicine, Macquarie University, Sydney, NSW, Australia; ^3^Centre for Research in Complex Systems, School of Community Health, Charles Sturt University, Albury, NSW, Australia; ^4^Electrical and Electronic Engineering Department, The University of Melbourne, Parkville, VIC, Australia

**Keywords:** type 2 diabetes mellitus, diabetic complications, diabetic neuropathy, genetic factors, uremic neuropathy, diabetic peripheral neuropathy, cardiac autonomic neuropathy

## Abstract

Type 2 diabetes mellitus (T2DM) is a global public health problem of epidemic proportions, with 60–70% of affected individuals suffering from associated neurovascular complications that act on multiple organ systems. The most common and clinically significant neuropathies of T2DM include uremic neuropathy, peripheral neuropathy, and cardiac autonomic neuropathy. These conditions seriously impact an individual’s quality of life and significantly increase the risk of morbidity and mortality. Although advances in gene sequencing technologies have identified several genetic variants that may regulate the development and progression of T2DM, little is known about whether or not the variants are involved in disease progression and how these genetic variants are associated with diabetic neuropathy specifically. Significant missing heritability data and complex disease etiologies remain to be explained. This article is the first to provide a review of the genetic risk variants implicated in the diabetic neuropathies and to highlight potential commonalities. We thereby aim to contribute to the creation of a genetic-metabolic model that will help to elucidate the cause of diabetic neuropathies, evaluate a patient’s risk profile, and ultimately facilitate preventative and targeted treatment for the individual.

## Introduction

Diabetes mellitus is a worldwide public health problem that currently affects over 382 million people globally, and by 2025 this number is expected to rise to approximately 592 million (1). In 60–70% of patients, diabetes is accompanied by neuropathies that can affect all organs of the body, and these complications are the key cause of morbidity and mortality among type 2 diabetes (T2DM) patients (2, 3). The mechanisms for the development of these neuropathies remain unclear, but are likely multifactorial and involve environmental and lifestyle factors, as well as genetic predisposition (4). This review is the first to consider the role of single and polygenetic influences in multiple diabetic complications associated with neuropathy, with the aim to look for common genes that drive the development and progression of diabetic neuropathies. Early subclinical detection of diabetic neuropathy, by assessing genetic predisposition as well as metabolic control and physiology of diabetic patients, is critical in facilitating early intervention and prevention of the potentially serious consequences of diabetic neuropathy (5, 6).

**Figure 1:**
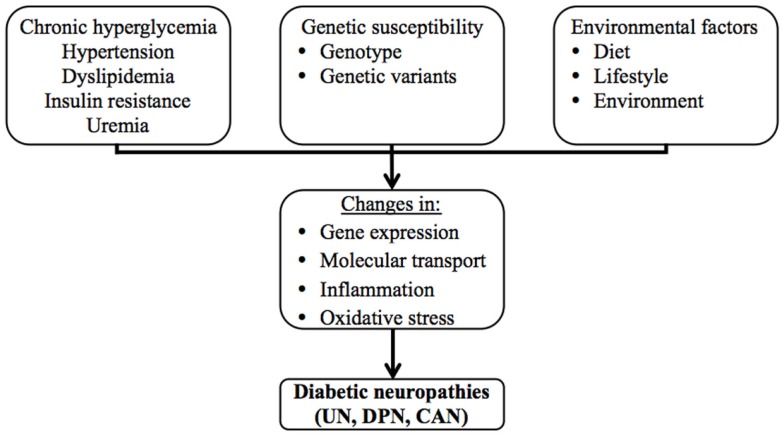
**The multifactorial etiology of the diabetic neuropathies**.

The clinically most significant diabetic neuropathies are uremic neuropathy (UN), diabetic peripheral neuropathy (DPN), and cardiac autonomic neuropathy (CAN). Although these neuropathies may remain asymptomatic for prolonged periods, they seriously impact the quality of life of the affected individuals and pose an immense burden on public health systems, both in terms of human and economic costs as they contribute to the need for dialysis and renal transplantation, physical disability and amputations, and cardiac interventions. Diabetic neuropathies have complex disease etiologies, which are still not completely understood. Chronic hyperglycemia, hypertension, dyslipidemia, insulin resistance, and uremia are thought to be the key factors that contribute to their pathogenesis, as they cause changes in gene expression, molecular transport, inflammation, and oxidative stress (Figure 1). Genetic susceptibility and environmental factors are also known to interact and contribute significantly to disease onset and progression, which is evident in observations of twin studies and familial clustering, and in the variation of the prevalence and presentation of these complications among individuals, and among different ethnic groups (7–10).

Recent advances in the application of genome-wide association studies (GWAS) and next-generation sequencing (NGS) are expected to expedite and enhance the ability to identify genetic variants associated with complex diseases, such as T2DM and its associated neuropathies. Studies conducted to identify genetic risk factors of diabetic neuropathies still remain scarce, but over 60 loci have already been identified to influence the risk of developing T2DM (11). Despite this effort, a significant amount of missing heritability remains to be explained as the majority of studies published in this field are limited by a lack of reproducibility and insufficient statistical power. While the search continues for novel rare variants that may help explain this missing heritability, it is evident that diabetes and its associated complications are the result of the combined effect of numerous genetic variants that may interact with each other and the environment to define disease risk, progression and severity. Complex interactions between risk factors contributing to the various diabetic neuropathies have been difficult to assess though, as the scope of diabetes-related genetic studies has often been limited to one particular candidate gene or one type of complication. GWAS conducted in this field so far have either focused on the risk of developing T2DM, or investigated risk factors of diabetes-associated retinal and renal vascular complications (12).

The pressing need to advance our understanding of the diabetic neuropathies is amplified by the rapidly increasing prevalence of T2DM and the associated increase in cardiovascular and kidney disease. UN and CAN remain underdiagnosed and there is an ongoing lack of effective treatment options for any of the diabetes-associated neuropathies. The main treatment approach for T2DM-associated neuropathies includes lifestyle changes and strict control of glucose and lipid levels, blood pressure control and addressing environmental factors (e.g., smoking), but these changes are often difficult to control, measure, or achieve and only have limited positive effects on slowing disease onset and progression (13–17). The only alternative treatment options are aimed at controlling pain and addressing the malfunctions of affected organs.

Diabetic neuropathy is a multifactorial disease that can affect the function of all organs of the body either directly or indirectly, mainly due to altered glucose metabolism, which affects neural tissue including central and peripheral nerves through oxidative stress mechanisms (18). Many of the genes and gene polymorphisms implicated in these complications to date are linked to glucose metabolism and biochemical changes, including inflammatory and immunological processes and hence may form a common genetic group that influences the development of diabetic complications. In order to allow us to target the diabetic neuropathies more effectively, future research needs to not only identify new genetic variants that contribute to each particular condition, but also to elucidate how such variants function and interact to give rise to the various diabetic neuropathies. By identifying commonalities, or differences, in the genes and proteins that determine the development and progression of the diabetic neuropathies, a deeper understanding of their complex disease etiologies will be gained, enabling the development of a more comprehensive approach to assessing an individual’s risk profile and ultimately providing personalized, targeted treatment. To contribute to the creation of an integrated genetic-metabolic model of diabetic neuropathies, this review provides an original overview of the diabetic neuropathies UN, DPN, and CAN in T2DM patients, and discusses the common genetic risk variants that have been associated with these disease complications.

## Clinical Presentation of Diabetic Neuropathies

### Uremic neuropathy

Approximately 30% of diabetic patients develop diabetic nephropathy (DN) and advanced renal failure, which are now the main cause of kidney failure in Western society (19). Diabetic nephropathy is also closely linked with the onset and progression of other diabetic neurovascular complications (20, 21). Advanced renal disease and renal failure lead to the release of uremic toxins. This uremia, combined with oxidative stress-related free radical activity, contributes to the damage of motor, sensory and autonomic nerves, making it a causative factor in the development of UN, DPN, and CAN (22, 23). In rare cases, the cranial nerves and pulmonary sympathetic nerves may also be affected (24–27). Approximately 60–100% of patients receiving dialysis treatment suffer from UN and the complication is more severe in diabetics than in non-diabetics (17, 28, 29). The most common form of UN is a distal symmetric polyneuropathy in the lower limbs, which is due to length-dependent axonal degradation and secondary focal loss of myelin sheaths (30, 31).

Uremic neuropathy remains largely asymptomatic until renal function has decreased by approximately 75%, when the glomerular filtration rate drops below 10–12 ml/min, which may not occur until 10–15 years after the onset of DN (Table 1). At this advanced stage of renal disease, the symptoms rapidly progress in severity over months as serum creatinine levels build up (32). Early sensory symptoms include paresthesia, paradoxical heat sensation and increased pain sensation in the lower extremities (33, 34). Restless leg syndrome and cramps are common, and symptoms may move proximally and spread to upper extremities (35). As the disease progresses, motor neuropathy becomes evident as weakness, impaired deep tendon reflexes, imbalance, numbness, and atrophy of the lower limbs. Approximately half of uremic patients also experience autonomic impairment, evident in postural hypertension, hyperhidrosis, and dysfunction of the digestive, excretory. and reproductive organs (17, 27, 36). However, clinical signs and symptoms due to UN, such as tingling in the feet, may be present much earlier (37).

**Table 1:** **Clinical presentation and management of the diabetic neuropathies**.

	Uremic neuropathy (UN)	Diabetic peripheral neuropathy (DPN)	Cardiac autonomic neuropathy (CAN)
Affected nerves	Distal symmetric polyneuropathy of sensory, motor and autonomic nerves including renal sympathetic nerves	Distal symmetric polyneuropathy of sensory and motor nerves. Distal sympathetic autonomic neuropathy may also be observed	Parasympathetic and sympathetic autonomic nerves of the heart and the vasculature
	In rare cases: cranial and pulmonary sympathetic nerves		
Pathogenesis	Exact mechanism unclear	Exact mechanism unclear	Exact mechanism unclear
	Advanced renal disease and build-up of uremic toxins are thought to affect neuron function	Hyperglycemia is thought to affect numerous pathways, causing nerve damage and inhibiting repair mechanisms	Hyperglycemia is thought to lead to accumulation of AGE products, which signal through their receptor RAGE, inducing chronic inflammation and nerve damage
Symptoms	Early sensory symptoms	Early sensory symptoms	Subclinical stage
	• Paresthesia	• Neuropathic pain	• Parasympathetic and sympathetic tone imbalance
	• Paradoxical heat sensation	• Sensitivity changes to vibration and temperature	• HRV changes
	• Increased pain sensation in lower extremities	• Numbness	• Alterations in baroreflex sensitivity
	• Restless leg syndrome		• Abnormalities in left ventricle
	• Cramps	Advanced disease motor-deficit symptoms	
		• Muscle weakness	Clinical stage
	Advanced disease motor-deficit symptoms	• Foot deformities	• Resting tachycardia
	• Muscle weakness	• Impaired deep tendon reflexes	• Exercise intolerance
	• Impaired deep tendon reflexes	• Imbalance	• Postural hypotension
	• Imbalance	• Changes in gait and postural sway	• Cardiac dysfunction
	• Numbness	Distal sympathetic autonomic neuropathy	• Diabetic cardiomyopathy
	• Atrophy of lower limbs	• Dry, cracked and warm skin
	Autonomic impairment	• Plantar calluses at weight-bearing areas
	• Postural hypotension
	• Hyperhidrosis
	• Dysfunction of digestive, excretory and reproductive organs
Diagnosis of somatic neuropathy	• Neurological assessment	• Physical examination of feet
	• Nerve conduction velocity testing	• Perception of vibration, temperature, pain
		• Ankle reflex
Diagnosis of autonomic neuropathy	• Foot muscle strength	
	• HRV	• Cardiovascular autonomic reflex tests	• Cardiovascular autonomic reflex tests
		• HRV	• HRV
Treatment	No preventative treatment	No preventative treatment	No preventative treatment
	Dialysis and renal transplantation	Intensive control of glycemia, lipidemia, hypertension and lifestyle changes reduce risk	Intensive control of glycemia, lipidemia, hypertension and lifestyle changes reduce risk

Due to the prolonged asymptomatic nature of the condition, diagnosis often does not occur until significant damage has already taken place. Detailed neurological assessment and nerve conduction velocity testing are the most accurate way of diagnosing UN, even in asymptomatic patients (32). Afferent and efferent nerves regulate kidney function, and it is the renal sympathetic nerves that play an important role in kidney function and kidney homeostasis via renorenal reflex pathways. In T2DM and other pathologies, abnormal sympathetic modulation of the kidney contributes significantly to abnormal renal function (38). Autonomic dysfunction can be evaluated by monitoring cardiovascular reflex and heart rate variability (HRV) (39, 40). Only dialysis and renal transplantation are known to reverse the effects of UN and improve neural function. Unfortunately these interventions are themselves associated with similar neuropathic complications (27, 30).

### Diabetic peripheral neuropathy

Approximately 50% of diabetic patients will develop DPN, which entails the risk of recurrent foot ulceration and potential amputation of lower extremities (Table 1) (41–44). The most common form of DPN is distal symmetrical polyneuropathy, which begins in the lower extremities and progresses to the upper limbs over time, giving rise to a “glove and stocking” pattern (5). Neuropathic pain, changes in sensitivity to vibration and thermal thresholds, as well as numbness is common characteristics of DPN (42). As the disease progresses, motor deficits, muscle weakness, and foot deformities may develop, affecting the individual’s gait (5, 45). The combined effects of peripheral neuropathy, foot deformities, changes in foot biomechanics, and impaired turning of all major joints, contributes to significant postural sway and enhanced fall risk in comparison to healthy individuals. DPN has also been associated with symptoms of distal sympathetic autonomic neuropathy, which may become evident as dry, cracked, and warm skin and the appearance of plantar calluses at weight-bearing areas (46–49). Physical examination of the feet for calluses and ulcers, and monitoring for changes in sensory perception and muscle strength of the feet, may provide an early indication of the somatic neuropathy of DPN. No preventative treatment exists, but strict metabolic control is thought to slow down DPN progression.

### Cardiac autonomic neuropathy

Cardiac autonomic neuropathy is thought to occur in 20–73% of T2DM patients, yet it often goes undiagnosed for several years (Table 1) (13). CAN alters cardiac rhythm through pathological changes, first occurring in the parasympathetic cardiac branches followed by sympathetic nervous system dysfunction, and thereby predisposes to cardiac arrhythmia (40, 50, 51). In some populations, it has been shown to increase the 5-year mortality risk by 50% (50, 52–54). The presence and severity of UN and DPN have been associated with earlier onset and higher prevalence of CAN, supporting the close epidemiological link between these complications, but the concurrent development of somatic and autonomic neuropathies has also been reported in T2DM, with CAN developing during the pre-diabetic condition (55–60). The exact pathogenesis of CAN remains unclear and our understanding of neural damage relies largely on models of somatic neuropathy (13, 48). Chronic hyperglycemia, dyslipidemia, and oxidative stress are thought to contribute to accelerated nerve damage, neuronal ischemia, and the dysfunction and apoptosis of neurons (13, 51, 61, 62). CAN may be subclinical for many years, while parasympathetic denervation occurs. This results in an imbalance between parasympathetic and sympathetic tone, leading to changes in HRV as a marker of sympathovagal balance, alterations in baroreflex sensitivity, and abnormalities of the left ventricle (2, 63–65). Within 5 years, CAN is thought to progress to sympathetic nerve damage, but the exact timeline is uncertain. In this clinical stage, patients experience resting tachycardia, exercise intolerance, postural hypotension, cardiac dysfunction, and diabetic cardiomyopathy (13).

Cardiovascular autonomic reflex tests can be used to monitor cardiac autonomic function by measuring changes in heart rate and blood pressure in response to deep respiration, the Valsalva maneuver and postural changes (47, 66, 67). New techniques for cardiac imaging and guidance on diagnostic procedures are expected to improve the detection of subclinical CAN (3, 13). Intensive control of glycemia, lipidemia, hypertension, and lifestyle changes may reduce the risk of CAN by up to 50%, but no therapeutic treatment exists that can prevent CAN completely (13, 62).

## Genetics of Diabetic Neuropathies

The commonalities in the clinical presentation and molecular pathways known to be involved in UN, DPN, and CAN support a close epidemiological link between these diabetic neuropathies, and the presence of one neuropathy frequently increases the risk of onset or severity of another (68, 69). Only a small number of studies have been carried out to investigate the genetic predisposition to these neuropathies in T2DM patients.

### Genetics of uremic neuropathy

Considering the increasing prevalence and severe implications of chronic kidney disease, it is not surprising that an extensive number of studies have been carried out to identify genetic variants that may contribute to renal disease, including DN, but far less is known of the genetics of UN. MalaCards, the human malady compendium database, lists four genes related to UN: *B2M*, *PTH*, *PSMC2*, and *PNRC1* (70). Beta-2-microglobulin (B2M) has been extensively studied in relation to diabetes, kidney disease, and cardiac events. In T2DM, it acts as a sensitive diagnostic marker for renal function; it is considered a uremic toxin and is also used as a surrogate marker for other uremic toxins (71–73). Elevated B2M plasma levels have been associated with cardiovascular disease and mortality in uremic patients (71). Parathyroid hormone (PTH) has also been implicated in several chronic complications of diabetes including retinopathy and nephropathy, as well as in renal disease in non-diabetics (74–76). It is considered a major uremic toxin, and variations in PTH levels have been associated with UN (77, 78). In a study conducted in non-diabetic dialysis patients, PTH levels were not found to be associated with autonomic dysfunction; to our knowledge this association has not been tested in diabetics (14, 79). Whether the genes identified by two GWAS linking DN with T2DM are also involved in UN is not clear (80–82). Statistically significant associations have been detected between DN in T2DM and single nucleotide polymorphisms (SNPs) in genes including *ACACB* (83), *ACE* (84), *ADIPOR2* (85), *AGER* (*RAGE*) (85), *AGTR1* (86), *AGT* (86), *AKR1B1* (87), *APOE* (88), *CCL5* (*RANTES*), and its receptor *CCR5* (89, 90), *CNDP1* (91), *CYBA* (92), *ELMO1* (93), *FABP-2* (94), *FRMD3* (95), *HSPG2* (85), *IL-6* (96), *KCNQ1* (97), *LPL* (88), *MMP9* (98), *MTHFR* (99), *MYH9* (100), *NCALD* (80, 101), *NOS3* (85), and *PPAR*γ*2* (102), *PVT1* (103), *SIRT1* (104), *SLC12A3* (80, 105), *VEGF* (106), rs1411766 (chromosome 13q) (107), rs1034589 (nearest gene: *RNF185*) (81), and several SNPs within the *LIMK2-SF1* region (81). Numerous candidate genes could be added to this list by including polymorphisms that have been shown to have a marginally significant association with DN, and gene expression studies that have associated DN with changes in expression levels of miRNAs and members of the NF-κB, TGF-β1 and complement pathways (19, 81, 85, 108–113).

### Genetics of diabetic peripheral neuropathy

Numerous studies have investigated genetic risk and protective factors for DPN in T2DM in particular, highlighting a potential association with specific polymorphisms in genes including *ACE* (114), *AKR1B1* (115), *ADRA2B* (116), *APOE* (117–119), *GPx-*1 (120), *IL-4* (121), *IL-10* (122), *IFN-*γ (122), *MTHFR* (123), *NOS1AP* (124), *NOS3* (125–127), *TLR4* (128), *UCP2* (129), and *VEGF* (Table 2) (130). Variants of mitochondrial genes have also been linked to T2DM, and recently polymorphisms in *ATPase 8*, *ND1*, *ND5*, and *MT-CYB* were suggested to affect DPN based on a study of a single individual (131). Further candidate genes have been proposed, based on their role in regulating DPN in type 1 diabetes (T1DM).

**Table 2:** **Genetic variants showing a significant association with diabetic neuropathies in T2DM**.

Gene	SNP/variant	Risk variant	Sample size	Ethnic group	Reference
**A. Diabetic peripheral neuropathy (DPN)**					
*ACE*	I/D (287 bp, intron 19)	D	1316 T2DM patients with DPN, 1617 controls	Caucasians and Asians	(114)
AKR1B1	−106 C/T	T	85 T2DM patients, 126 non-diabetic controls	Finnish	(115)
	(CA)*n* repeat (Z allele, *n* = 24)	Z-2			
Alpha 2B-AR	I/D	D	130 T2DM patients with DPN, 60 T2DM patients without DPN	Greek	(116)
APOE	rs429358 T/C, rs7412 C/T	C/C	158 T2DM patients	Japanese	(117)
GPx-1	rs1050450 C/T	T	1155 T1DM and T2DM patients	Caucasian	(120)
IL-4	VNTR (P1/P2 allele)	P1 allele	227 T1DM and T2DM patients with DPN, 241 non-diabetic controls	Turkish	(121)
IL-10	−1082 G/A	G	198 T2DM patients, 202 non-diabetic controls	South Indian	(122)
IFN-γ	874 A/T	A	198 T2DM patients, 202 non-diabetic controls	South Indian	(122)
MTHFR	677 C/T	T	230 T1DM and T2DM patients with DPN, 282 Non-diabetic controls	Turkish	(123)
NOS1AP	rs1963645 A/G	G	26 Diabetic patients with CKD	White American	(124)
	rs6659759 T/C	C	21 Non-diabetics with CKD		
	rs16849113 C/T	T	30 Diabetic patients with CKD	African	
	rs880296 C/G	G	20 Non-diabetics with CKD	American	
NOS3	27VNTR (4a/b)	4a	353 T2DM patients with DPN	North and South Indian	(125)
	−786 T/C (rs270744)	C	905 T2DM patients without DPN		
TLR4	896 A/G (rs4986790)	AA	246 T1DM patients, 530 T2DM patients	German Caucasian	(128)
	1196 C/T	CC			
UCP2	−866 G/A	A	197 T2DM patients	Japanese	(129)
VEGF	I/D (18bp, at −2549)	D	84 T2DM patients with DPN, 90 Non-diabetic controls	Romanian	(130)
**B. Cardiac autonomic neuropathy (CAN)**					
*CHT1*	SNP in 3′UTR	T	95 T2DM patients, 208 non-diabetic controls	African Americans	(132)
TCF7L2	rs7903146 C/T	T	154 T2DM patients, 185 non-diabetic controls	Italian	(9)
	rs7901695 T/C	C			
TCF-α	−308 A/G	A	eight diabetic patients with congestive heart failure, three healthy controls	Serbian	(133)

*Several genes and their genetic variants have been associated with diabetic peripheral neuropathy (DPN, A) and cardiac autonomic neuropathy (CAN, B) in type 2 diabetes mellitus (T2DM). This table lists the first publications to identify a statistically significant association between these genetic variants and the diabetic neuropathy, or refers to a recent meta-analysis if too many contradictory publications were found on the topic. bp, base pair; CKD, chronic kidney disease; SNP, single nucleotide polymorphism; T1DM, type 1 diabetes mellitus; T2DM, type 2 diabetes mellitus; UTR, untranslated region*.

### Genetics of cardiac autonomic neuropathy

Studies on genetic variants involved in CAN remain scarce and potential candidate genes are often selected based on their implication in somatic peripheral neuropathies. A number of genes have been linked to CAN in particular, based on their involvement in autonomic dysfunction in animal models and in human patients of T1DM or other cardiac complication, or due to their involvement in inflammatory responses that are thought to contribute to neuronal injury. These candidate genes include *ACE* (134), *ADRB2* (135), *AKR1B1* (47, 136), *APOE* (137), *CAT* (138), *GNAS* (139), *MTHFR* (140), *NF-*κ*B* (141), *NOS* (142, 143), *TLR2* and *TLR4* (144), and *SREBP-1* (145). The only genetic polymorphisms that have been associated with the risk of CAN in T2DM patients are *TCF7L2* (9), *TNF-*α (133, 146), and *CHT1* (Table 2) (132). Serum levels of interleukin 6 (IL-6) (147, 148) and adiponectin (ADIPOQ) (146) have also shown to change with CAN in T2DM patients, but no genetic variants have been linked to this observation. The heritability of CAN-related parameters has been associated particularly with variants of the *ACE* gene, but twin studies in non-diabetic subjects have produced contradictory results with regards to the effect size imposed by genetic and environmental factors on CAN risk (10, 149–151).

### Common genetic risk factors

**Figure 2:**
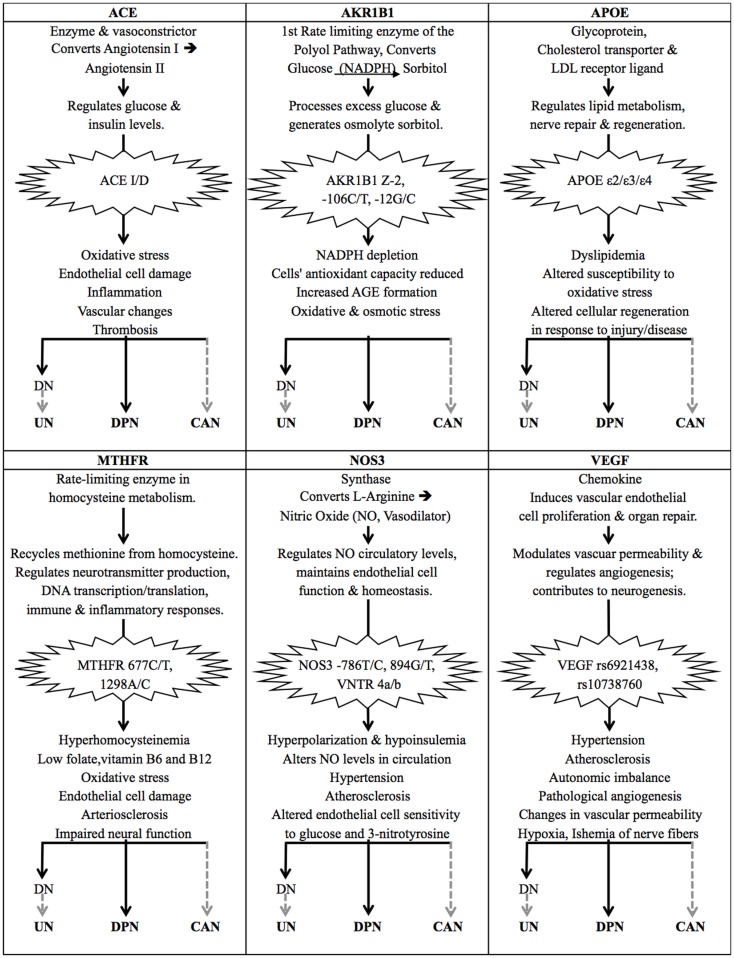
**The effects of common genetic variants implicated in the diabetic neuropathies**. The gene products of *ACE*, *AKR1B1*, *APOE*, *MTHFR*, *NOS3*, and *VEGF* are all involved in important molecular pathways that have been associated with type 2 diabetes (T2DM) and diabetic neuropathies. Certain genetic variants of these genes have been identified as risk factors for diabetic nephropathy (DN) and diabetic peripheral neuropathy (DPN) in particular. DN causes renal damage and a build-up of uremic toxins, which is thought to lead to uremic neuropathy (UN), suggesting that these genetic risk factors also affect the onset and progression of UN. The same genetic variants have also been implied in diabetic autonomic neuropathy and cardiac complications, suggesting their involvement in CAN, but their direct association with CAN in T2DM patients remains to be proven. Solid black arrows indicate that a statistically significant association has been published; dashed gray arrows indicate a suspected association.

This review highlights the lack of studies conducted to investigate the genetic risk factors that contribute to diabetic neuropathies, particularly UN, DPN, and CAN. Based on the publications identified in this review, polymorphisms within *ACE*, *AKR1B1*, *APOE*, *MTHFR*, *NOS3*, and *VEGF* have been shown to contribute to DPN as well as DN. Considering the functions of these genes, it is possible that these same variants also contribute to UN and CAN (Figure 2). If one also considers candidate genes that have been implied in but not significantly associated with the diabetic neuropathies, this list of potentially common risk factors could be extended significantly. The implication of these candidate genes comes as no surprise as all of them are associated with key molecular pathways that have been linked to diabetes and its complications (48, 50, 152). This observation warrants further investigation into how exactly these genetic variants may contribute to the diabetic neuropathies, and what their pharmacogenetic effects may be.

#### *ACE* 

The *ACE* gene encodes the angiotensin-converting enzyme, which is a key component of the renin–angiotensin system. It is a potent vasoconstrictor that converts angiotensin I to angiotensin II, and is involved in inducing proteolysis of the vasodilator bradykinin 2 (153–155). Angiotensin II is attributed a role in the regulation of glucose and insulin levels, and the hyperglycemia-induced increase in angiotensin II levels has been linked to diabetes risk and found to induce oxidative stress, damage to endothelial cells, inflammation, vascular changes, and thrombosis (Figure 2) (156–158). The insertion/deletion (I/D) polymorphism of a 287 base pair Alu sequence in intron 16 of the *ACE* gene is of particular interest, as this polymorphism alone is thought to be responsible for 46% of the variance of the ACE enzyme present in serum (159, 160). The deletion variant D, and the DD genotype in particular, has been associated with significantly higher plasma levels of ACE (161, 162). In DPN, the *ACE* I/D polymorphism has been identified as a genetic risk factor (Table 1) (114, 163). No studies have investigated the association between *ACE* and UN, but the renin–angiotensin system and its component angiotensin II are thought to contribute to most pathological processes involved in DN and therefore may also be contributing to UN (164, 165). This is supported by several meta-analyses that suggest that the D allele is a risk factor for DN (166–168); the I/I genotype is thought to fulfill a protective function in this regard (169). The *ACE* I/D polymorphism alone, and haplotypes including this and other markers within the *ACE* gene, have been associated with DN progression and end stage renal disease in particular, which in turn leads to UN (84, 170–172). The *ACE* D/D genotype has also been linked with impaired circadian blood pressure variation in T2DM, which is associated with autonomic neuropathy, hypertension, and limited kidney function (134, 173). Studies investigating the association between the *ACE* I/D polymorphism and diabetic complications have produced very contradictory results, providing a good example of an issue encountered in the majority of genetic studies of T2DM risk factors. These contradictions have been attributed to inter-ethnic variation of allele distribution, and a lack of consideration for haplotypes and linkage disequilibrium that exists between different genetic variants across the *ACE* gene (172, 174).

The *ACE* I/D variant is also thought to affect a patient’s response to therapeutics commonly used in diabetes: angiotensin-converting enzyme inhibitors (ACEIs) and angiotensin receptor blockers (ARBs) are used to treat hypertension and numerous diabetic complications including retinopathy, nephropathy, neuropathy, CAN, and cardiovascular disease (175–181). The polymorphism has also been reported to affect the efficiency of statins used to treat hypercholesterolemia (182). Contradictory results have been published on either the II or DD genotype facilitating a better treatment outcome and due to these inconsistencies, and a lack of sufficient genetic data, no consensus has been reached on the benefit of routinely genotyping patients for the *ACE* I/D polymorphism (176, 182–187).

#### *AKR1B1* 

Aldose reductase is the first rate-limiting enzyme in the polyol pathway, which reduces glucose to sorbitol in an NADPH-dependent reaction. In hyperglycemic conditions, an increased flux of glucose through the polyol pathway has been shown to contribute to diabetic neurovascular complications by depleting NADPH levels and reducing a cell’s antioxidant capacity by increasing the formation of advanced glycation end products (AGEs), and contributing to oxidative and osmotic stress (Figure 2) (188). Polymorphisms within the aldose reductase gene, *AKR1B1*, and its promoter region have been linked to diabetic neuropathies and microvascular complications (115, 189). These include the (CA)*n* microsatellite (*n* = 22–29; Z allele *n* = 24) located approximately 2.1 kb upstream of the gene’s transcription start site, and two polymorphisms in the basal promoter region at −106 C/T (rs759853) and −12 G/C. The (CA)*n* Z-2 and the −106 C/T polymorphisms have been found to be closely linked (115). Patients suffering from diabetic neurovascular complications have been shown to express higher levels of aldose reductase; a haplotype including the microsatellite Z-2 and SNP −106 C variants has been associated with the highest expression levels of *AKR1B1* and is thought to pose a high risk for diabetic microvascular complications (190). In T2DM patients with DN, the Z-2 and −106T alleles have been shown to have a synergistic effect on increasing the risk of patients suffering cardio-renal endpoints (191, 192). The overexpression of *AKR1B1* has not only been reported in glomeruli, but also in the peripheral nerves of T2DM patients, where it is thought to contribute to the disease pathology of diabetic neuropathy (193, 194). T2DM patients with the *AKR1B1* −106 T/T genotype have been reported to experience more severe neuropathological deterioration in the sural and radial nerves, contributing to the onset of DPN (115). The Z-2 variant has not been found to significantly affect DPN to date (115, 195). *AKR1B1* variants have been associated with CAN in T1DM patients, but no association has been shown in individuals with T2DM (47, 195, 196).

Aldose reductase inhibitors (ARIs) have been researched over many years for their use in treating diabetic complications, but although animal studies have provided promising results, clinical trials in search of an optimal ARI for humans continues (197–206). Only Epalrestat is currently on the market in Japan to treat DPN-associated symptoms (198, 207, 208). Epalrestat has also been reported to delay progression of diabetic retinopathy, nephropathy, and neuropathy, and has been suggested as a treatment for CAN (207–209). As polymorphisms of *AKR1B1* affect aldose reductase expression levels, it is suspected that these polymorphisms may affect a patient’s optimum dosage of ARIs, but this remains to be investigated.

#### *APOE* 

The apolipoprotein E (apoE) gene product is a cholesterol transport protein and a major ligand of the low density lipoprotein (LDL) receptor, which plays an important role in lipid metabolism, hypercholesterolemia, as well as nerve repair and regeneration (Figure 2) (210, 211). The *APOE* gene has three main alleles, which are the result of two SNPs that cause amino acid changes at 112 Cys/Arg (rs429358 C/T) and 158 Arg/Cys (rs7412 T/C). The ε3 variant is the most common and has the wild type alleles at both SNPs; the ε4 allele has a substitution giving rise to an arginine at amino acid 112, and the least common allele, ε2, has a substitution leading to a cysteine at amino acid 158 (212). These alleles give rise to the apoE2, E3, and E4 protein isoforms, which differ in charge and stability, and their roles in various disease pathologies. The ε2 allele is thought to result in higher circulatory levels of apoE, but lower levels of LDL cholesterol. It has been shown to be defective in binding the LDL receptor, but has been suggested to protect against micro- and macro-vascular complications in T2DM (213, 214). The ε4 variant has been associated with lower levels of circulatory apoE, but higher plasma levels of total cholesterol, LDL, and VLDL (very low density lipoprotein), and has been associated with higher risk and severity of neuropathological diseases (119, 214–216). Contradictory results have been obtained with regards to the role of *APOE* variants in diabetes, which may be due to population-specific effects and variations in study designs (210). *APOE* polymorphisms have been linked to DN risk, but no definite conclusion has been made on whether the ε2 or ε4 allele confers disease risk (217). The *APOE* variants have been implicated in neurological disorders of the central and peripheral nervous system, yet results on their role as a risk or protective factor in DPN have also been inconsistent and a recent meta-analysis could not establish a definite association (119, 212). Several studies have shown that the *APOE* ε4 allele may be associated with particularly severe DPN (117–119). Interestingly, the *APOE* ε2 allele was associated with lower levels of DPN in most published studies, and may have a protective role in neuromuscular diseases (118, 119, 212, 218). Based on the association of *APOE* and its ε4 allele with disorders of the nervous system, as well as observations made in *APOE*(−/−) knockout mice, it is considered a candidate gene for CAN risk, but no association has been published (50, 137, 219).

Pharmacogenetic effects of the *APOE* genotype have also been found in response to therapeutic approaches used in diabetes, hyperlipidemia, and neurodegenerative diseases (220–223). Metformin has been found to enhance *APOE* expression, which is known to affect peripheral nerve regeneration (220, 224, 225); if this effect is isoform specific is unclear. *APOE* genetic variants have been reported to respond differently to diet therapy and lipid-lowering therapy using statins. Some studies suggest that carriers of the ε4 isoform are less responsive to statins, but more responsive to diet and lifestyle interventions. However controversial results have also been published and no consensus has been reached with regards to routine *APOE* genotyping (163, 210, 221, 223, 226–229).

#### *MTHFR* 

The 5,10-methylene-tetrahydrofolate reductase (MTHFR) enzyme and its genetic variants have been implicated in various disease pathologies. The two most studied missense mutations, 677 C/T (rs1801133) and 1298 A/C (rs1801131), have been associated with T2DM and diabetic neuropathies, independently and in synergy (230). Both polymorphism leads to a reduced enzyme activity of MTHFR, which has been linked to hyperhomocysteinemia (elevated plasma levels of homocysteine) and lower levels of folate, and vitamins B6, and B12 (230–233). At least 35% of this variation in homocysteine levels is thought to be due to folate and vitamin B12 levels, and a change in diet may counteract the negative effects of the 677 C/T polymorphism to some extent (234, 235). This indicates that diet is a confounding factor that should be considered in *MTHFR* studies. Hyperhomocysteinemia has been shown to induce oxidative damage of endothelial cells, arteriosclerosis, and impaired neural function (236, 237), and has been associated with neurovascular diabetic complications (238, 239). The 677 C/T variant has been linked to DPN (123, 237, 240). Albuminuria, glomerular lesions, and DN in T2DM have also been linked to hyperhomocysteinemia and the 677 T allele, but no direct association with UN has been made (237, 241–243). Elevated homocysteine level is also considered a risk factor for diabetic autonomic neuropathy (244). No studies investigated the role of *MTHFR* polymorphisms in relation to CAN specifically, but in a non-diabetic study, the 677 T allele was linked to lower HRV than the CC genotype, which is a diagnostic indicator of CAN (140).

Mutations of the *MTHFR* gene have been found to affect a patient’s response to numerous drugs, including metformin, nitrous oxide anesthesia, methotrexate, and chemotherapy (245–249). In diabetics, metformin treatment has been linked to vitamin B12 deficiency; this vitamin deficiency may in turn cause hyperhomocysteinemia, which is exacerbated in patients with the 677 C/T mutation and increases the risk of DPN, diabetic retinopathy, and vascular thrombosis (250–252). In the presence of the 677 C/T and 1298 A/C polymorphisms of *MTHFR*, treatment with MTHFR-5, vitamins (such as folic acid) and minerals are suggested to supplement the deficient enzyme (246, 251).

#### *NOS3* 

Nitric oxide (NO) is produced from L-arginine by endothelial nitric oxide synthase (NOS3 or eNOS) and functions as a vasodilator that is essential for the maintenance of endothelial cell function and homeostasis (Figure 2) (253). Genetic variants of *NOS3* have been linked to changes in circulating levels of NO and have been implicated in hypertension, atherosclerosis, and diabetic microvascular complications (254). Three polymorphisms within this gene have been of particular interest as they have been shown to alter NOS3 enzyme activity and plasma levels of NO: the −786 T/C (rs2070744) and 894 G/T (rs1799983) SNPs, as well as the variable number tandem repeat (VNTR) polymorphism within intron 4 that can give rise to two deletion variants, 4a and 4b. The regulation of *NOS3* is complicated by strong linkage that exists between several SNPs within the gene, as well as potential interactions of these alleles with other genetic variants (255). Numerous studies have linked these three polymorphisms of *NOS3* and related haplotypes with the development and progression of DN (256, 257). No association with UN has been made, but NO and the nitric oxide synthases are known to fulfill important functions in the peripheral nervous system. Several recent studies have implicated *NOS3* variants in DPN. Particularly the *NOS3* VNTR 4a/b variant is suspected of having a haplotype-dependent effect on diabetic microvascular complications and diabetic neuropathy (258). Many contradictory results have been reported on the role of *NOS3* variants in relation to diabetic neuropathies as they seem to be significantly influenced by population-specific effects (125–127). Nitric oxide is also an important regulator of the autonomic nervous system and the *NOS3* −786 T/C variant has been found to contribute to autonomic imbalance in various disease pathologies, but it has not yet been associated with CAN in T2DM in particular (143).

The association of NOS3 with neuropathic pain and inflammation makes it an interesting therapeutic target (259). As the *NOS3* polymorphisms discussed here affect NOS3 enzyme activity as well as NO plasma levels, they may need to be considered during drug design and dosage. L-arginine supplementation has been tested to restore NO levels in diabetes, but contradictory results about the effectiveness of this NO precursor have been published and whether *NOS3* polymorphisms have an effect on this supplementation remains unclear (260–263).

#### *VEGF* 

The vascular endothelial growth factor (VEGF) is a chemokine that induces vascular endothelial cell proliferation and organ repair. It plays a key role in modulating vascular permeability and regulating angiogenesis, and numerous studies have highlighted its positive contribution to neurogenesis (264, 265). A significant level of heritability has been found to affect VEGF levels in circulation, up to 60−80%, of which 50% are determined by its SNPs rs6921438 and rs10738760 (266–269). Although several studies suggest that an upregulation of VEGF may fulfill a protective and neurotropic function, and that administration of VEGF may improve diabetic neuropathy, other studies show that an increase in circulating VEGF levels is associated with pathological angiogenesis and changes in vascular permeability, thereby contributing to cardiovascular and diabetic complications, ischemia of nerve fibers and hypoxia (Figure 2) (48, 270–274). In DN, the contradictory effects of VEGF may be explained by VEGF potentially fulfilling different functions during disease onset and progression, and in diabetics versus non-diabetics (275). *VEGF* polymorphisms have been linked to the risk of developing DN, but no significant association could be detected between *VEGF* polymorphisms and UN or DPN so far (106, 276).

Anti-VEGF antibodies, or antibody fragments, are currently only being used to treat diabetic retinopathy and macular edema (277, 278). Studies in non-diabetic patients with age-related macular degeneration have suggested that polymorphisms of VEGF contribute to the variability in response to anti-VEGF treatment, but contradictory results have been published (279–286). With regards to neuropathy treatment, gene transfer of VEGF is being investigated (287, 288).

## Conclusion

Type 2 diabetes mellitus is associated with the risk of developing debilitating and life-threatening neurovascular complications including UN, DPN, and CAN. While recent advances in genetic research tools have already provided us with a wealth of data on genetic variants that may affect an individual’s risk of developing diabetes and its associated retinal and renal vascular complications, little research has been conducted in the area of diabetic neuropathies. As new NGS developments drive the search for rare genetic variants that may explain the missing heritability of T2DM, it is important to not lose sight of the need to further investigate the variants we have already identified. How do these genetic variants function and interact in the various diabetic neuropathies? How can we use this knowledge to assess an individual’s risk of diabetic neuropathy? And ultimately, how can we apply this information to provide patients with targeted, personalized treatment? Most studies of T2DM genetics investigate polymorphisms and single gene effects in the diabetic population in general, irrespective of the patients’ complications, or they only focus on one diabetic complication in particular. Yet, we are already aware of common risk factors contributing to the various diabetic complications. Recent reviews by Tang et al. (111) and Forbes & Cooper (152) have provided a comprehensive overview of the genetic and molecular pathways contributing to T2DM and its vascular complications, highlighting commonalities between the different conditions. To our knowledge, our review is the first to provide an overview of common genetic variants that contribute to the diabetic neuropathies, including UN, DPN, and CAN.

Extensive research in the areas of chronic kidney disease, peripheral neuropathy, and atherosclerosis has given rise to a substantial list of genetic variants that may be associated with these conditions, paving the way for research into their role in diabetic neuropathy. This review suggests that polymorphism within *ACE*, *AKR1B1*, *APOE*, *MTHFR*, *NOS3*, and *VEGF* may act as common genetic risk factors for the diabetic neuropathies in T2DM (Figure 2). Numerous other genetic variants have been implicated, yet not significantly associated with all of UN, DPN, and CAN. Polymorphisms in genes of the inflammatory and thrombotic pathways, as well as the plasma levels of the resulting gene products including, CD40 ligand and MCP-1, may be of particular interest to future studies, as these processes have been implicated in the development of nerve damage in several diabetic neuropathies and have also been linked to other diabetic complications including ischemic stroke (289–291). More research is required, especially in the field of UN, to elucidate the possible synergistic effects of gene products. There may be common genetic variants associated with both CAN and UN, which may be difficult to disentangle as both CAN and UN have a pre-clinical phase that can be present early in diabetic disease progression and even at the pre-diabetic stage. The identification of genetic risk factors may be further complicated by the fact that genes that play a role in the diverse biochemical pathways associated with the development of diabetic neuropathy, including oxidative stress, AGE products and the polyol pathway, may be selectively turned on or off depending on the stage of disease progression.

Another interesting area of research, with regards to the genetics of diabetic neuropathies, is the field of pharmacogenetics. Treatment options for gene related products is an important new direction for therapy, which is gaining momentum with the identification of gene variants being associated with diabetic complications. It is important to note that genetic polymorphisms, such as the ones discussed in this review, may modify a patient’s response to medication. Further genetic data will be essential to verify the possible benefits of routine genotyping, and the pharmacogenetics that may guide personalized medication, dosage and delivery.

In addition to increasing research efforts with regards to the diabetic neuropathies, methodological issues experienced by genetic studies also need to be tackled. When one reviews the literature published on genetic risk factors of T2DM and the diabetic complications, major issues such as the lack of reproducibility and insufficient statistical power are very apparent. These shortcomings can be largely attributed to small samples sizes, population-specific effects and the significant heterogeneity between study designs. The generation of standards and guidelines on the definition and diagnosis of the different neuropathies, and a better understanding of what confounding factors or comorbidities must be taken into account, will hopefully reduce this variation and improve our ability to generate more reproducible results. Taking into consideration the effect of haplotypes, linkage disequilibrium, and gene-environment interactions may also explain a significant part of this variation. By reducing the heterogeneity between studies, meta-analysis would be more applicable and could help increase the statistical power by combining smaller datasets. Advances in NGS are also expected to enhance the statistical power of studies, but the use of sufficiently large sample sizes continues to be paramount, as many studies still include less than 100 subjects. Inter-ethnic differences in allele frequencies have also been identified for most genetic variants, which points toward the need for population-specific studies. Ultimately, even when genetic variants alone do not indicate a significant association with a condition, associated changes in gene expression and regulation may still identify the variant’s product as a useful biomarker. Genetic variants should not be viewed in isolation; and neither should the diabetic neuropathies.

To further our understanding of UN, DPN, and CAN in T2DM, we suggest that whole genome analysis will be required to investigate not only patients presenting with single, but also with multiple neuropathies. This will allow the identification of commonalities and differences in risk and protective factors of these diabetic complications. The study of haplotypes and gene interactions with lifestyle or environmental factors will lead to a better understanding of disease etiologies. The use of populations with a limited genetic pool, such as the Arab Bedouins, in which consanguineous marriages are common and family sizes are large, could also enhance our understanding of the heritability of T2DM and the diabetic neuropathies (292).

By highlighting the commonalties in genetic risk variants in the diabetic neuropathies, this review aims to stress the need of a unified genetic model for these diabetic complications. We cannot expect genetics alone to provide a full explanation for the cause and progression of the diabetic neuropathies, but research is continuing to support its important role in these multifactorial complications (85, 111, 293–296). Ultimately, our understanding of the complex disease etiology of these diabetic neuropathies will require a multi-system multifactorial approach, combining genetics, epigenetics, proteomics, and treatment effects. A better disease process model would allow individualized preventative care and targeted drug delivery, and have a serious impact on improving patients’ lives and reducing healthcare costs. The complexity of T2DM does not only lie in finding the missing puzzle pieces, but also in putting them together to see the ‘big picture’.

## Author Contributions

The idea for this review was conceived by HJ and further developed by all contributing authors. IW researched and wrote the manuscript. Expert advice and editing on the topic of diabetes-associated neuropathies was provided by HJ, AK, SL, and KK; HA provided advice and edited sections on the topic of genetics. All authors contributed to revising the final version of the manuscript for accuracy.

## Conflict of Interest Statement

The authors declare that the research was conducted in the absence of any commercial or financial relationships that could be construed as a potential conflict of interest.

## Supplementary Material

The Supplementary Material for this article can be found online at http://journal.frontiersin.org/article/10.3389/fendo.2015.00088

## Abbreviation

ACEI, angiotensin-converting enzyme inhibitor; AGE, advanced glycation end-product; ARB, angiotensin receptor blocker; ARI, aldose reductase inhibitor; CAN, cardiac autonomic neuropathy; DN, diabetic nephropathy; DPN, diabetic peripheral neuropathy; GWAS, genome-wide association study; HRV, heart rate variability; I/D, insertion/deletion; LDL, low density lipoprotein; NGS, next generation sequencing; NO, nitric oxide; SNP, single nucleotide polymorphism; T1DM, type 1 diabetes mellitus; T2DM, type 2 diabetes mellitus; UN, uremic neuropathy; VLDL, very low density lipoprotein; VNTR, variable number tandem repeat.
