# Supplementary material for: Identifying Common Genetic Risk Factors of Diabetic Neuropathies
Source: Front Endocrinol (Lausanne). 2015 May 28;6:88. doi: 10.3389/fendo.2015.00088 (PMC4447004; doi:10.3389/fendo.2015.00088)
Supplement: Supplementary file 1 [file Table_1.PDF]

Supplementary Material

Identifying Common Genetic Risk Factors of Diabetic Neuropathies

Ini-Isabée Witzel[1*], Herbert F. Jelinek[2, 3], Kinda Khalaf[1], Sungmun Lee[1], Ahsan H. Khandoker[1,4] and Habiba Alsafar[1].

[1]Biomedical Engineering Department, Khalifa University of Science, Technology and Research, Abu Dhabi, UAE
[2]Australian School of Advanced Medicine, Macquarie University, Sydney, NSW, Australia
[3]Centre for Research in Complex Systems and School of Community Health, Charles Sturt University, Albury, NSW, Australia
[4] Electrical and Electronic Engineering Department, The University of Melbourne, Parkville, VIC, Australia

*** Correspondence:** Dr. Ini-Isabée Witzel (IIW)**,** Biomedical Engineering Department**,** Khalifa University of Science, Technology and Research, PO Box 127788, Abu Dhabi, UAE.
Ini.Witzel@gmail.com

1. Supplementary Table

Supplementary Table 1. Gene symbols used throughout review.

Gene Symbol	Description
ACACB	Acetyl-Coenzyme A Carboxylase Beta
ACE	Angiotensin I Converting Enzyme
ADIPOQ	Adiponectin
ADIPOR2	Adiponectin Receptor 2
ADRA2B	Adrenoreceptor Alpha 2B
ADRB2	Adrenoreceptor Beta 2
AGER (RAGE)	Advanced Glycosylation End Product-Specific Receptor
AGT	Angiotensinogen
AGTR1	Angiotensin II Receptor, Type I
AKR1B1	Aldose Reductase 1
APOE	Apolipoprotein E
ATPase 8	Mitochondrially Encoded ATP Synthase 8
B2M	Beta-2-Microglobulin
CAT	Catalase
CCL5	Chemokine (C-C motif) Ligand 5
CCR5	Chemokine (C-C motif) Receptor 5
CHT1 (SLC5A7)	Solute Carrier Family 5 Member 7
CNDP1	Carnosine Dipeptidase 1
CYBA	Cytochrome b-245, Alpha Polypeptide

ELMO1	Engulfment and Cell Motility 1
FABP-2	Fatty Acid Binding Protein 2, Intestinal
FRMD3	FERM3 Domain Containing 3
GNAS	Guanine Nucleotide Binding Protein G, Alpha Stimulating Activity Polypeptide 1
GPx-1	Glutathione Peroxidase 1
HSPG2	Heparan Sulfate Proteoglycan 2
IFN-γ	Interferon Gamma
IL-4	Interleukin 4
IL-6	Interleukin 6
IL-10	Interleukin 10
KCNQ1	Potassium Voltage-gated Channel, KQT-like Subfamily, Member 1
LIMK2	LIM domain Kinase 2
LPL	Lipoprotein Lipase
MMP9	Matrix Metallopeptidase 9
MT-CYB	Cytochrome b
MTHFR	Methylenetetrahydrofolate Reductase
MYH9	Myoxin, Heavy chain 9
Nav1.7	Voltage-gated Sodium Ion Channel
NCALD	Neurocalcin Delta
ND1	Mitochondrially Encoded NADH Dehydrogenase 1
ND5	Mitochondrially Encoded NADH Dehydrogenase 5
NF-κB	Nuclear Factor kappa B
NOS	Nitric Oxide Synthase
NOS1AP	Nitric Oxide Synthase 1 (neuronal) Adaptor Protein
NOS3	Nitric Oxide Synthase 3 (endothelial)
PNRC1	Proline-rich Nuclear Receptor Coactivator 1
PPARγ2	Peroxisome Proliferator-Activated Receptor γ
PSMC2	Proteasome 26S Subunit, ATPase, 2
PTH	Parathyroid Hormone
PVT1	Pvt1 Oncogene (long intergenic non-protein coding RNA 79)
RNF185	Ring Finger Protein 185
SF1	Splicing Factor 1
SIRT1	Sirtuin 1
SLC12A3	Solute Carrier Family 12 (Sodium/Chlorine Transporter), Member 3
SREBP-1 (SREBF1)	Sterol Regulatory Element Binding Protein-1
TCF7L2	Transcription Factor 7-like 2
TGF-β	Tumor Growth Factor β
TLR2	Toll-Like Receptor 2
TLR4	Toll-Like Receptor 4
TNF-α	Tumor Necrosis Factor α
UCP2	Uncoupling Protein 2
VEGF	Vascular Endothelial Growth Factor
